# The Association Between Adverse Childhood Experiences (ACEs), Bullying Victimization, and Internalizing and Externalizing Problems Among Early Adolescents: Examining Cumulative and Interactive Associations

**DOI:** 10.1007/s10964-023-01907-2

**Published:** 2023-12-08

**Authors:** Nora Trompeter, Alexander Testa, Julia H. Raney, Dylan B. Jackson, Abubakr A. A. Al-shoaibi, Kyle T. Ganson, Iris Yuefan Shao, Jason M. Nagata

**Affiliations:** 1 Institute of Psychiatry, Psychology & Neuroscience, King’s College London, London, UK; 2 Department of Management, Policy and Community Health, University of Texas Health Science Center at Houston, Houston, TX USA; 3 Department of Pediatrics, Division of Adolescent and Young Adult Medicine, University of California, San Francisco, San Francisco, CA USA; 4 Department of Population, Family, and Reproductive Health, Johns Hopkins Bloomberg School of Public Health, Baltimore, MD USA; 5 Factor-Inwentash Faculty of Social Work, University of Toronto, Toronto, ON Canada

**Keywords:** Adverse childhood experiences, Bullying, Peer victimization, Psychopathology

## Abstract

Both adverse childhood experiences (ACEs) and bullying victimization are linked with mental health problems in adolescents. However, little is known about the overlap between the two factors and how this impacts adolescent mental health problems (i.e., internalizing and externalizing problems). The current study analyzed data from 8,085 participants (47.7% female; 44.1% racial/ethnic minority) in the Adolescent Brain Cognitive Development (ABCD) study, baseline (2016–2018, ages 9–10 years) to Year 2. Regression analyses were used to estimate associations between ACEs, bullying victimization and mental health problems, respectively, adjusting for sex, race/ethnicity, country of birth, household income, parental education, and study site. The findings showed that both ACEs and bullying victimization were independently associated with higher internalizing and higher externalizing problems. However, no significant interaction was found between ACEs and bullying victimization. Overall, the results align with the cumulative risk model of adversity, linking cumulative ACEs and bullying victimization to internalizing and externalizing problems in early adolescents.

## Introduction

Adverse childhood experiences (ACEs) are defined as potentially traumatic or otherwise negative events in a child’s social or familial environment (e.g., child abuse, neglect) that may cause distress or harm and disrupt the child’s development and well-being (Felitti et al., 1998). Prevalence estimates suggest that approximately 60.1% of people experience at least one ACE during their childhood and 16.1% experience four or more ACEs (Madigan et al., 2023). ACEs have been consistently linked with poor mental health outcomes in adolescents, reflecting a dose-dependent relationship whereby more ACEs are linked with poorer mental health (Green et al., 2010). The original ACEs were based on seminal work by Felitti et al. (1998) and focused on areas of abuse and neglect, as well as household dysfunction (e.g., parental alcohol abuse). Building on these original ACEs, scholars have been working to redefine and update the original ACEs to reflect other relevant adversities that impact the mental health of young people (Finkelhor et al., 2013). Such work has suggested that bullying victimization may be considered an ACE (Karatekin & Hill, 2019). Bullying refers to repeated, intentional acts of interpersonal aggression with an imbalance of power (Olweus, 1991). This could include verbal threats, physical harm, and social exclusion both in-person and online (cyberbullying; (Smith et al., 2008)). Like the original ACEs, bullying victimization is linked to mental health problems, and potentially long-lasting effects (Brunstein Klomek et al., 2019). This study builds on emerging evidence suggesting that ACEs and bullying victimization co-occur, by investigating how this co-occurrence impacts mental health problems, especially during early adolescence.

The developmental trajectory from childhood to early adulthood is a crucial period, as it is vulnerable to the establishment of several health-related risk factors and development of mental health problems (Solmi et al., 2021). From a developmental perspective, ACEs are commonly experienced during early childhood or persistently throughout childhood (Fisher et al., 2010). In contrast, issues with bullying victimization are more common during late childhood and early adolescence, as social contexts change throughout development and children begin to spend more time with their peers (Hawes et al., 2021). Thus, understanding the interplay between ACEs and bullying victimization on mental health problems in early adolescents is critical for informing early interventions. Previous research on contextual factors relating to bullying found that several family characteristics such as harsh parenting (Barker et al., 2008), low family income (Barker et al., 2008), and child maltreatment place children at greater risk for bullying victimization (Bowes et al., 2009). As such, it is important to further investigate the association between ACEs and bullying victimization to understand whether both are independently associated with mental health problems, or whether having a history of ACEs amplifies the negative association between bullying victimization and mental health problems.

The cumulative risk model is the most prominent theoretical framework to understand the impact of ACEs. The cumulative risk model proposes that cumulative adversity is linearly associated with negative outcomes, whereby more adversity is linked with more negative outcomes (Evans et al., 2013). Research tends to support this model (Appleyard et al., 2005), whereby the accumulation of toxic stress generated by ACEs over time is thought to explain the dose-dependent association between ACE exposure and mental health outcomes (Shonkoff et al., 2012). However, the model has also been criticized as it implies that all negative events are equal and that all potential combinations have the same outcome (Jaen et al., 2023). Previous research has shown that some ACEs (e.g., childhood sexual abuse) are linked with worse outcomes than others (e.g., parental separation), and that there are interactive effects between different combinations of ACEs (Putnam et al., 2020).

While interactive effects have been observed among the traditional ACEs, whereby some combinations have a multiplicative effect on mental health problems (i.e., experiencing more adversities does not just increase the risk for mental health problems linearly, but proliferates the risk), this has not been extended to bullying victimization. It is unclear whether bullying victimization would have a cumulative effect, as predicted by the cumulative risk theory, or a multiplicative effect on mental health problems in adolescents. Here, a cumulative effect refers to both the exposure to ACEs and exposure to bullying victimization being associated with more mental health problems in a dose-dependent relationship whereby greater exposure is linked with worse outcomes. A multiplicative effect would see an interaction of the ACEs and bullying victimization whereby one might alter the direction of the relationship with mental health problems such that it amplifies the problem. For example, having been exposed to ACEs may amplify the relationship between bullying victimization and mental health problems. One study to date has investigated the overlap between bullying victimization and substance use in adolescents (Afifi et al., 2020). Findings support the cumulative risk model, whereby both ACEs and bullying victimization were uniquely associated with substance use without interacting with one another. However, no such research has yet explored mental health problems at a broader scale, including internalizing problems (i.e., depression, anxiety), in the context of early adolescence.

Understanding whether ACEs and bullying victimization follow a cumulative or multiplicative pattern will have important implications for both the conceptualization of ACEs and early intervention. According to the cumulative risk model, current conceptualizations of ACEs partly rely on the understanding that ACEs are interrelated and cumulative in explaining outcomes. If the overlap between ACEs and bullying victimization is cumulative in terms of mental health problems, this will provide further evidence for including bullying victimization in screening tools for ACEs. However, if the overlap is multiplicative ACEs and bullying victimization may be better understood, and screened for, as separate risk factors for mental health problems.

## Current Study

The current study aimed to examine the relationship between ACEs, bullying victimization and mental health problems (internalizing and externalizing problems) in early adolescents. Specifically, this study aimed to examine whether the overlap between ACEs and bullying victimization is cumulative or interactive regarding both internalizing and externalizing problems. Based on prior research, it was hypothesized that both exposure to ACEs and bullying victimization would be associated with higher internalizing and externalizing problems. No a-priori hypotheses were made regarding the interaction between ACEs and bullying victimization. Instead, two competing hypotheses were examined, the cumulative risk hypothesis and the multiplicative risk hypothesis. The cumulative risk model would suggest that both exposures (ACEs and bullying victimization) are independently associated with greater mental health problems (significant main effects), with no significant interaction. In contrast, the multiplicative risk model would suggest that exposure to ACEs interacts with exposure to bullying victimization, whereby the relationship between bullying victimization and mental health problems is greater among adolescents who have experienced ACEs compared to adolescents who have not experienced ACEs.

## Methods

### Participants and Procedure

Secondary data analyses were conducted using data from baseline through 2-year follow-up of the Adolescent Brain Cognitive Development (ABCD) study (4.0 release). Details regarding the ABCD study participants, recruitment, protocol, and measures have previously been described (Barch et al., 2018). The ABCD study is a longitudinal cohort study (baseline 2016–2018) of physical and mental health, neurological, and cognitive development across the U.S., from 21 different recruitment sites. Baseline data collection took place when participants were aged 9–10 years. After omitting participants with missing outcome data, 8085 children remained in the analytic sample. Institutional review board (IRB) approval was received from the University of California, San Diego as well as the respective IRBs of each study site. All participants provided written assent, and written informed consent was obtained from their caregivers.

### Measures

#### ACE score

The ABCD study assesses eight of ten ACEs reflecting the items in the original CDC-Kaiser ACE study (Felitti et al., 1998): physical abuse, sexual abuse, household violence, household mental illness, substance abuse in the household, divorce/separation, emotional neglect, and physical neglect. This coding scheme is consistent with prior research in the ABCD study (Raney et al., 2022), and align with recent recommendations for research on ACEs to investigate a variety of different models, including a cumulative approach, to further our understanding and accurately capture the complexity of ACEs and their link with mental health problems (Jaen et al., 2023).

A cumulative ACE was calculated by summing yes response to any of the eight ACEs by either parent or child at any time point (baseline, 1-year follow-up, or 2-year follow-up). The total ACE score was categorized to 0, 1, 2, 3, and ≥4, as a cumulative ACE score of ≥4 has documented greater risk concentration at this threshold (Anda et al., 2020), and is in line with previous research in the ABCD study cohort (Nagata et al., 2022). Previous research has indicated that using a cumulative score is supported by data in population studies converging to one unidimensional factor (Lian et al., 2022)

#### Bullying victimization

At the 2-year follow-up adolescents completed two measures on bullying victimization: cyberbullying victimization (Stewart et al., 2014) and traditional bullying victimization (Prinstein et al., 2001). Cyberbullying victimization was assessed with the single-item question, *“Have you ever been cyberbullied, where someone was trying on purpose to harm you or be mean to you online, in texts, or group texts, or on social media (like Instagram or Snapchat)?”*. Traditional bullying victimization was assessed using the Peer Experiences Questionnaire (Prinstein et al., 2001). Questions cover domains of overt (e.g., “*A kid threatened to hurt or beat me up”*), relational (e.g., “*A kid left me out of what they were doing*”), or reputational victimization (e.g., “*A kid tried to damage my social reputation by spreading rumors about me”*) on a 5-point Likert scale ranging from 1 (“*Never*”) to 5 (“*A few times a week*”). For the purpose of this study, a dichotomized bullying victimization score based on both questionnaires (*“no bullying victimization”* versus *“any bullying victimization”)* was used for the main analyses. For exploratory analyses on cumulative bullying experiences, a separate cumulative victimization score was created to represent exposures to different types of bullying based on the four domains measured (i.e., cyberbullying, overt bullying, relational bullying, and reputational bullying).

#### Mental health problems

To examine adolescents’ mental health problems, data from the parent-reported Child Behavior Checklist (Achenbach, 1999) at the 2-year follow-up was used. The measure is widely used to capture dimensional psychopathology symptoms and contains two broad summary scores (internalizing and externalizing problems). Internalizing problems summarize scores on the Anxious/Depressed (e.g., *Fears mistakes*), Withdrawn/Depressed (e.g., *Lacks energy*), and Somatic Complaints scores (e.g., *Stomach aches*). Externalizing problems summarize the Rule-Breaking Behavior (e.g., *Breaks rules*) and Aggressive Behavior scores (e.g., *Gets in fights*). In line with scoring guidelines, total scores were recorded as z-scores with higher scores reflecting more internalizing/externalizing problems.

#### Covariates

Several socio-demographic covariates were considered in the current study. These include participants’ sex at birth (male or female), race/ethnicity (Non-Latino/Hispanic White, Non-Latino/Hispanic Black, Native American, Latino/Hispanic, Asian, or Other), and country of birth (born in U.S. or outside U.S.), which were reported at baseline by parents. Parents also reported highest parent education and household income at Year 2. Highest parent education was categorized as high school or lower versus college or higher. Household income was grouped into two categories reflecting the U.S. median household income: less than $75,000 and $75,000 or more (Semega et al., 2019).

### Missing Data

Of the 11,855 participants, 62% had complete data available on the variables of interest and covariates. It was initially planned to use a complete-case analysis, however, due to the relatively high level of missing data, multiple imputation was used instead. Most participants had missing data on the mental health problems measure (*n* = 3877; 32.6%), which was treated as the outcome measure and not imputed as no appropriate auxiliary variables were identified. Missing data on the remaining variables was imputed through multivariate imputation using chained equations (MICE) with 20 imputed datasets using information from the predictors and covariates. Sensitivity analyses were conducted using complete-case analysis, as well as full multiple imputation with potential auxiliary variables. No differences were detected and hence, the findings from the partial multiple imputation analyses are reported. Full results are reported in Supplementary 1.

### Data Analyses

The analysis plan was preregistered on the Open Science Framework. All deviations from the a priori analytic plan are explicitly described and explained below. All analysis code and the preregistered analysis plan are available at https://osf.io/862hu/?view_only=c9e8dca0fd084b52949b46f86e3a4196. Data analyses were performed using Stata 17. Linear regression models were used to examine the association between cumulative ACEs, bullying victimization, and internalizing and externalizing problems, respectively. Firstly, regression models with two main effects tested whether ACEs and bullying victimization had an independent, cumulative association with mental health problems. Secondly, the interaction between ACEs and bullying victimization on internalizing and externalizing problems respectively was examined by adding an interaction term to the regression models. All models adjusted for sociodemographic covariates and data collection site. Propensity weights were applied to match key sociodemographic variables in the ABCD Study to the American Community Survey from the U.S. Census (Heeringa & Berglund, 2020).

In addition to the pre-registered analyses, exploratory analyses were conducted to examine the dose-dependent relationship of bullying victimization using a cumulative bullying variable. All analyses were repeated as above.

## Results

### Sample Characteristics

The sociodemographic characteristics of the included participants are outlined in Table 1. The analytic sample was approximately balanced according to sex and was racially and ethnically diverse. Experiences of ACEs and bullying victimization were common in the sample. In terms of bullying victimization, relational bullying was most common (*n* = 5339, 66.1%), followed by reputational bullying (*n* = 2930, 36.3%), overt bullying (*n* = 2457, 30.4%), and cyberbullying (*n* = 710, 8.8%).

**Table 1:** Sociodemographic and ACE characteristics of included and excluded participants

Sociodemographic characteristics	Frequency/Mean	Percentage/SD
Sex		
Female	3854	47.69%
Male	4228	52.31%
Age (in years) at baseline (mean, SD)	11.96	(0.65)
Race/ethnicity at baseline		
White	4504	55.90%
Latino	1350	16.76%
Black	1363	16.92%
Asian	483	5.99%
Native American	284	3.52%
Other	73	0.91%
Country of birth (%)		
U.S.	7856	97.18%
Outside of U.S.	228	2.82%
Household income (%) at Year 2		
Less than $75,000	4724	63.15%
$75,000 and greater	2757	36.85%
Parents’ highest education (%) at Year 2		
College education or more	6878	85.21%
High school education or less	1194	14.79%
Bullying victimization (%) at Year 2		
No bullying victimization	2141	26.51%
Any bullying victimization	5934	73.49%
ACEs total (%) at Year 2		
0	1544	19.10%
1	2929	36.23%
2	2279	28.19%
3	1029	12.73%
4+	304	3.76%
CBCL internalizing score (mean, SD)	47.77	(10.50)
CBCL externalizing score (mean, SD)	44.48	(9.80)

Raw data is presented

Totals may not add up to the total sample size due to missing data.

*SD* standard deviation, *CBCL* Child behavior checklist

### ACES and Bullying Victimization

**Fig. 1:**
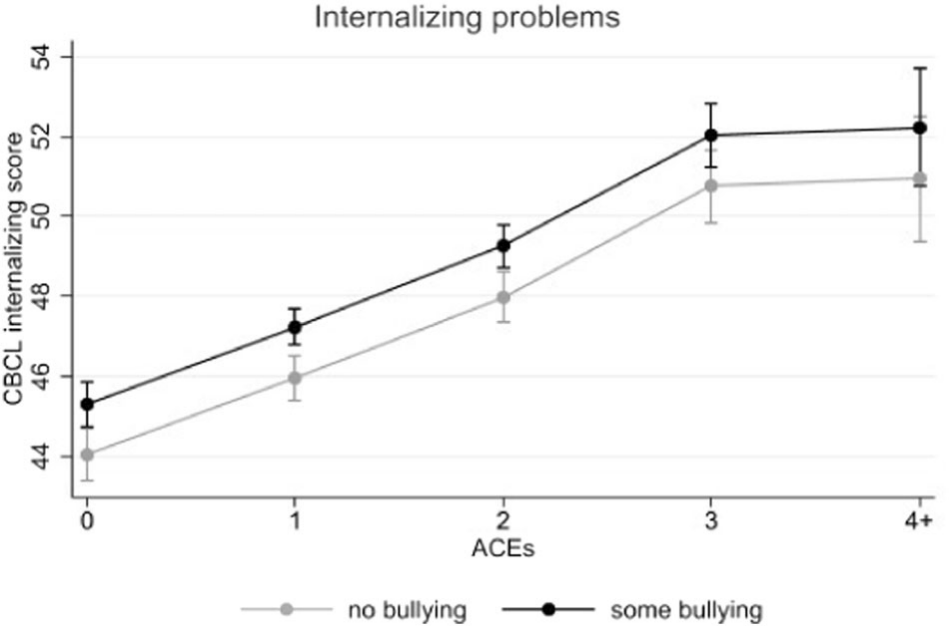
Associations between ACEs and bullying victimization regarding internalizing problems with 95% confidence intervals

**Fig. 2:**
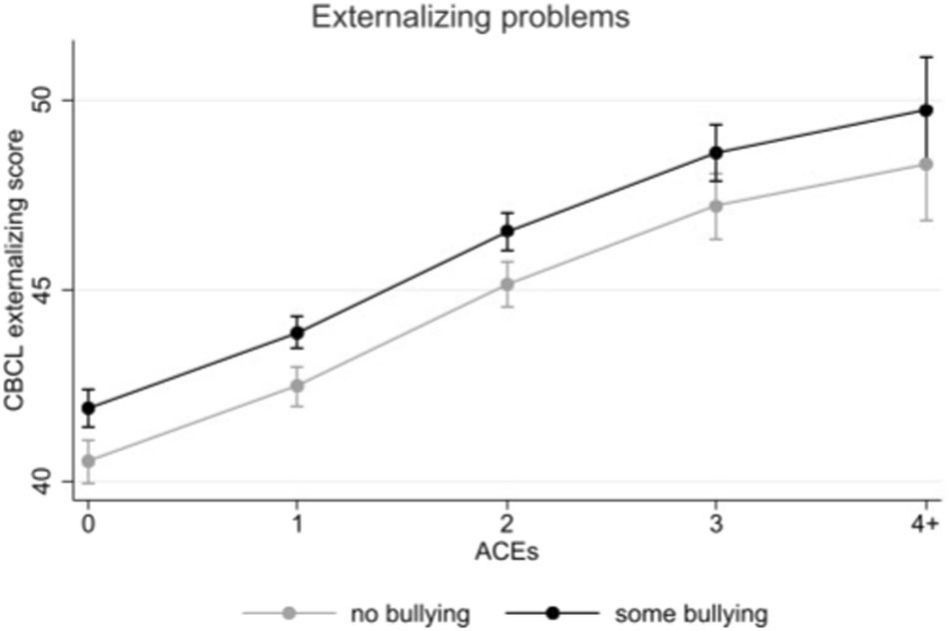
Associations between ACEs and bullying victimization regarding externalizing problems with 95% confidence intervals

Both experiences of ACEs and bullying victimization were independently associated with higher internalizing and higher externalizing problems (see Table 2). Notably, ACEs showed a cumulative pattern with more ACEs being linked with higher internalizing and externalizing problems. No significant interactions were found between ACEs and bullying victimization (B = 0.23 [−0.37; 0.87], *p* = 0.458; B = 0.33 [−0.21; 0.87], *p* =0.227), see Figs. 1, 2.

**Table 2:** Coefficients and 95% confidence intervals (CIs) for the main effect models (*N* = 8085)

	Internalizing problems		Externalizing problems	
	B (95% CI)	*p*	B (95% CI)	*p*
ACEs total				
0	Reference			
1	**1.92 (1.27–2.58)**	**<0.001**	**1.98 (1.39–2.57)**	**<0.001**
2	**3.95 (3.22–4.67)**	**<0.001**	**4.65 (3.98–5.31)**	**<0.001**
3	**6.72 (5.76–7.69)**	**<0.001**	**6.69 (5.80–7.59)**	**<0.001**
4+	**6.91 (5.33–8.50)**	**<0.001**	**7.80 (6.30– 9.30)**	**<0.001**
Bullying victimization				
No bullying victimization	Reference			
Any bullying victimization	**1.28 (0.72–1.84)**	**<0.001**	**1.40 (0.88 −1.93)**	**<0.001**

Bold indicates *p* < 0.05. ABCD propensity weights were applied based on the American Community Survey from the US Census. Adjusted models include sex, race/ethnicity, country of birth, household income, parent education, and study site. “Reference” indicates the reference category for categorical variables

In addition to our pre-registered analyses, ACEs were also testd individually (see Supplementary 2). Sexual abuse, household abuse, parental substance use, and parental mental health difficulties were independently associated with higher internalizing and externalizing problems, whereas physical neglect was only associated with higher externalizing problems. No significant associations were found for emotional neglect, physical abuse, or parental separation. However, results should be interpreted with caution as large confidence intervals for the analysis indicate low statistical power and limited confidence. As such, no interaction analyses were conducted with individual ACEs given the large statistical power required for such analyses (Blake & Gangestad, 2020).

### Exploratory Analyses

Both experiences of ACEs and cumulative bullying victimization were independently associated with higher internalizing and higher externalizing problems (see Table 3). No significant interaction was found between ACEs and cumulative bullying victimization in terms of internalizing problems (*p* = 0.128), see Supplementary 2. However, a significant interaction was found for externalizing problems (*p* = 0.013), whereby the difference in externalizing problems between adolescents with multiple bullying exposures compared to adolescents with fewer bullying exposures was more pronounced at higher ACE exposures, see Supplementary 2.

**Table 3:** Coefficients and 95% confidence intervals (CIs) for the main effect models examining cumulative bullying

	Internalizing problems		Externalizing problems	
	B (95% CI)	*p*	B (95% CI)	*p*
ACEs total				
0	Reference			
1	**1.88 (1.22–2.53)**	**<0.001**	**1.92 (1.34–2.51)**	**<0.001**
2	**3.78 (3.06–4.50)**	**<0.001**	**4.44 (3.79–5.10)**	**<0.001**
3	**6.40 (5.44–7.35)**	**<0.001**	**6.28 (5.41–7.16)**	**<0.001**
4+	**6.51 (4.93– 8.09)**	**<0.001**	**7.28 (5.81–8.75)**	**<0.001**
Bullying victimization				
0	Reference			
1	−0.06 (−0.71–0.60)	0.864	−0.25 (−0.84–0.34)	0.402
2	**1.30 (0.58–2.02)**	**<0.001**	**1.52 (0.84–2.20)**	**<0.001**
3	**2.86 (2.02–3.68)**	**<0.001**	**3.20 (2.24–3.37)**	**<0.001**
4	**4.42 (2.99–5.84)**	**<0.001**	**5.52 (4.10–6.94)**	**<0.001**

Bold indicates *p* < 0.05. ABCD propensity weights were applied based on the American Community Survey from the US Census. Adjusted models include sex, race/ethnicity, country of birth, household income, parent education, and study site. “Reference” indicates the reference category for categorical variables

## Discussion

Currently, little is known about how the co-occurrence of ACEs and bullying victimization is associated with mental health problems in early adolescents. In line with previous research, findings showed strong and independent associations between both ACEs and bullying victimization, with mental health problems (Afifi et al., 2020). Overall, the findings provide further evidence for a cumulative risk model of adversity (McLaughlin et al., 2012).

Similar to previous research on ACEs, both internalizing and externalizing problems showed similar patterns of associations (Brieant et al., 2022). Both exposure to ACEs and bullying victimization may place adolescents at risk for any mental health problem, rather than specific symptoms. Indeed, similar underlying mechanisms may link ACEs and bullying victimization to mental health problems, such as emotion dysregulation (Herd & Kim-Spoon, 2021), low self-esteem (Kim et al., 2022),and maladaptive coping (Trompeter et al., 2018). However, to date, such research has largely focused on specific types of ACEs or bullying victimization. Based on findings from the current study, future research should consider both cumulative ACEs and bullying victimization to further understand the underlying psychological mechanisms linking these exposures to mental health problems. While the current study was framed within the cumulative risk model of adversity, other models and approaches should be tested to determine how to best conceptualize co-occurring adversities throughout childhood. For example, future research should examine how bullying victimization fits within the threat-deprivation model of adversity, which posits that adversities have differential impacts on development depending on whether they align with a threat-response (e.g., family conflict) or a deprivations-response (e.g., emotional neglect) (McLaughlin & Sheridan, 2016).

While the findings support a cumulative risk pattern overall, one exception regarding cumulative bullying victimization and externalizing problems should be noted. That is, among adolescents with ACE exposure there was a greater difference in externalizing problems between adolescents with high cumulative bullying exposure and low cumulative bullying exposure compared to those with no ACE exposure (see Figure 4). Adolescents who have a history of ACE exposure may be at increased risk for externalizing problems if they also experience bullying victimization. While the finding was small in effect size, this finding has important implications for early intervention efforts targeting externalizing problems that typically onset in adolescence, such as substance use (Behrendt et al., 2009). Such intervention efforts may benefit from targeting early adolescents who have experienced both ACEs and bullying victimization to reduce externalizing problems to avoid escalation throughout adolescence.

The present study findings have significant policy, clinical, and public health implications, particularly for screening and prevention efforts. ACEs affect early adolescents at a critical time of development, and there have been ongoing calls for screening during primary care visits (Pardee et al., 2017). Findings from the current study suggest that in addition to screening for the original ACEs in pediatric care settings for example, screening for bullying victimization may improve detection of adversity and more accurately identify adolescents at risk for mental health problems. Schools and educators should be aware of the overlap between ACEs and bullying victimization and consider that adolescents who have experienced ACEs and/or bullying victimization are likely to be experiencing other types of victimization and/or mental health problems. In particular, while educators may not have insight into specific childhood adversities faced by students, they might have knowledge about general adversity. However, more research is needed to understand potential protective factors that might mitigate the association between ACEs, bullying victimization and adolescent mental health problems.

Despite these important implications, several limitations should be noted. Firstly, due to the observational nature of the study, no causal relationships can be made, and residual confounders may exist; however, known confounders were adjusted for. Secondly, questions regarding ACEs and bullying victimization were retrospective and hence vulnerable to recall bias and social desirability bias. Lastly, our conceptualization of bullying victimization was broad by including any instances of bullying victimization. This was in part due to the nature of the cyberbullying measure, which did not capture the frequency of bullying. Future research should examine whether different patterns hold when examining chronic bullying victimization.

## Conclusion

To date, little was known about the unique relationship between ACEs and bullying, with mental health problems in early adolescents. The current study found further evidence for the cumulative risk model of adversity, linking cumulative ACEs and bullying victimization to internalizing and externalizing problems in early adolescents. Future research and current screening practices for childhood adversity should consider including bullying victimization as a form of childhood adversity to enhance our understanding of childhood adversity’s impacts on mental health problems and improve early detection of childhood adversity.

### Supplementary Information

Supplementary Information

Supplementary Information

Supplementary Information
