# Supplementary material for: The Association Between Adverse Childhood Experiences (ACEs), Bullying Victimization, and Internalizing and Externalizing Problems Among Early Adolescents: Examining Cumulative and Interactive Associations
Source: J Youth Adolesc. 2023 Dec 8;53(3):744–52. doi: 10.1007/s10964-023-01907-2 (PMC10838217; doi:10.1007/s10964-023-01907-2)
Supplement: Supplementary file 1 — Supplementary Information [file 10964_2023_1907_MOESM1_ESM.docx]

*Table S3.* Coefficients and 95% confidence intervals (CIs) for the main effect models examining individual ACEs.

|  | Internalizing problems | | Externalizing problems | |
| --- | --- | --- | --- | --- |
|  | B (95% CI) | *p* | B (95% CI) | *p* |
| ACEs | | | | |
| Emotional neglect | 0.15 (-2.13 – 2.44) | .895 | 0.83 (-1.22 – 2.87) | .429 |
| Physical neglect | 1.00 (-0.08 – 2.06) | .068 | **1.53 (0.49 – 2.57)** | **.004** |
| Physical abuse | 0.18 (-2.66 – 3.01) | .902 | 0.55 (-2.49 – 3.57) | .722 |
| Sexual abuse | **3.28 (0.50 – 6.05)** | **.021** | **4.52 (1.67 – 7.38)** | **.002** |
| Household abuse | **1.99 (1.46 – 2.51)** | **<.001** | **3.66 (3.19 – 4.13)** | **<.001** |
| Parental substance abuse | **0.98 (0.45 – 1.52)** | **<.001** | **1.14 (0.65 – 1.64)** | **<.001** |
| Parental mental health difficulties | **4.17 (3.57 – 4.78)** | **<.001** | **3.22 (2.67 – 3.78)** | **<.001** |
| Parental separation | 0.39 (-0.37 – 1.14) | .317 | -0.28 (-0.96 – 0.40) | .420 |
| Bullying victimization | | | | |
| No bullying victimization | Reference | | | |
| Any bullying victimization | **1.23 (0.67 – 1.80)** | **<.001** | **1.38 (0.87 -1.90)** | **<.001** |

*Note.* Bold indicates p<0.05. ABCD propensity weights were applied based on the American Community Survey from the US Census. Adjusted models include sex, race/ethnicity, country of birth, household income, parent education, and study site. “Reference” indicates the reference category for categorical variables.
