# Supplementary material for: The Association Between Adverse Childhood Experiences (ACEs), Bullying Victimization, and Internalizing and Externalizing Problems Among Early Adolescents: Examining Cumulative and Interactive Associations
Source: J Youth Adolesc. 2023 Dec 8;53(3):744–52. doi: 10.1007/s10964-023-01907-2 (PMC10838217; doi:10.1007/s10964-023-01907-2)
Supplement: Supplementary file 2 — Supplementary Information [file 10964_2023_1907_MOESM2_ESM.docx]

**Complete case analysis**

Table S1. Regression results from the main effect models (N=7,443).

|  | Internalizing problems | | Externalizing problems | |
| --- | --- | --- | --- | --- |
|  | B (95% CI) | *p* | B (95% CI) | *p* |
| ACEs total | | | | |
| 0 | Reference | | | |
| 1 | **1.69 (1.01- 2.38)** | **<.001** | **1.99 (1.37 – 2.61)** | **<.001** |
| 2 | **3.57 (2.82 – 4.31)** | **<.001** | **4.47 (3.79 – 5.14)** | **<.001** |
| 3 | **6.57 (5.58 – 7.56)** | **<.001** | **6.88 (5.95 – 7.81)** | **<.001** |
| 4+ | **5.82 (4.42 – 7.43)** | **<.001** | **7.17 (5.60 – 8.74)** | **<.001** |
| Bullying victimization | | | | |
| No bullying victimization | Reference | | | |
| Any bullying victimization | **1.21 (0.64 – 1.79)** | **<.001** | **1.43 (0.88 -1.97)** | **<.001** |

*Note.* Bold indicates p<0.05. ABCD propensity weights were applied based on the American Community Survey from the US Census. Adjusted models include sex, race/ethnicity, country of birth, household income, parent education, and study site.

**Multiple imputation of independent and dependent variables**

Table S2. Regression results from the main effect models (N=11,871).

|  | Internalizing problems | | Externalizing problems | |
| --- | --- | --- | --- | --- |
|  | B (95% CI) | *p* | B (95% CI) | *p* |
| ACEs total | | | | |
| 0 | Reference | | | |
| 1 | **1.78 (1.09- 2.47)** | **<.001** | **1.90 (1.25 – 2.55)** | **<.001** |
| 2 | **3.94 (3.17 – 4.71)** | **<.001** | **4.62 (3.93 – 5.30)** | **<.001** |
| 3 | **6.62 (5.71 – 7.52)** | **<.001** | **6.53 (5.65 – 7.41)** | **<.001** |
| 4+ | **6.83 (5.39– 8.27)** | **<.001** | **7.69 (6.32 – 9.05)** | **<.001** |
| Bullying victimization | | | | |
| No bullying victimization | Reference | | | |
| Any bullying victimization | **1.32 (0.78 – 1.86)** | **<.001** | **1.40 (0.91 -1.89)** | **<.001** |

*Note.* Bold indicates p<0.05. ABCD propensity weights were applied based on the American Community Survey from the US Census. Adjusted models include sex, race/ethnicity, country of birth, household income, parent education, and study site.
