# Supplementary material for: The Association Between Adverse Childhood Experiences (ACEs), Bullying Victimization, and Internalizing and Externalizing Problems Among Early Adolescents: Examining Cumulative and Interactive Associations
Source: J Youth Adolesc. 2023 Dec 8;53(3):744–52. doi: 10.1007/s10964-023-01907-2 (PMC10838217; doi:10.1007/s10964-023-01907-2)

Supplementary 2

*Figure 3.* Associations between ACEs and bullying victimization regarding internalizing problems with 95% confidence intervals.


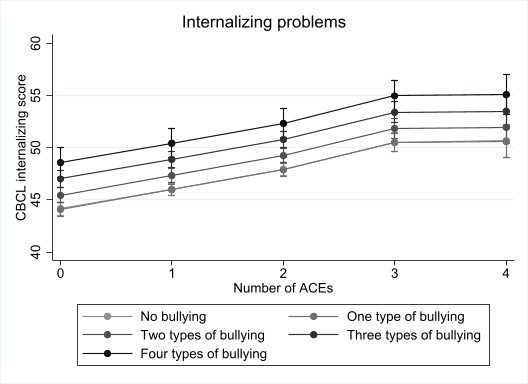


*Figure 4.* Associations between ACEs and bullying victimization regarding externalizing problems with 95% confidence intervals.


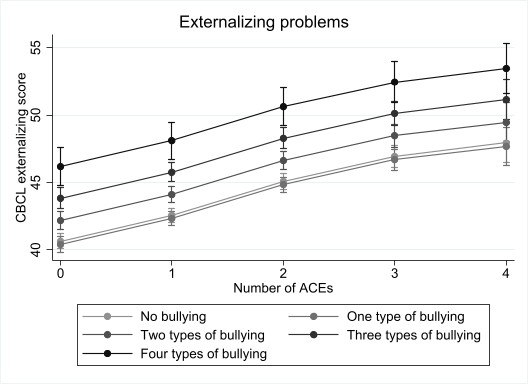

Supplement: Supplementary file 3 — Supplementary Information [file 10964_2023_1907_MOESM3_ESM.docx]
